# Different measures of fundamental frequency and vocal satisfaction among transgender men and women

**DOI:** 10.1590/2317-1782/e20240087en

**Published:** 2025-01-27

**Authors:** Diego Henrique da Cruz Martinho, Eric Rodrigues Dias, Ana Carolina Constantini

**Affiliations:** 1 Departamento de Saúde Interdisciplinaridade e Reabilitação, Faculdade de Ciências Médicas, Universidade Estadual de Campinas – UNICAMP - Campinas (SP), Brasil.; 2 Programas de Residência Multiprofissional, Hospital das Clínicas, Faculdade de Medicina, Universidade de São Paulo – USP - São Paulo (SP), Brasil.

**Keywords:** Voice, Voice Training, Speech Acoustics, Gender Identity, Communication

## Abstract

**Purpose:**

To verify possible correlations between f_o_ and voice satisfaction among Brazilian transgender people.

**Methods:**

An observational, cross-sectional quantitative study was conducted with the Trans Woman Voice Questionnaire (TWVQ), voice recording (sustained vowel and automatic speech) and extraction of seven acoustic measurements related to f_o_ position and variability in transgender people. Participants were divided into two groups according to gender. After descriptive and inferential analysis, comparison between both groups was performed by Student’s t-test and the correlation between f_o_ measurements and the TWVQ protocol was calculated by Pearson’s correlation (p<0.05).

**Results:**

A total of 11 transgender women (mean age = 26.91) and seven transgender men (mean age = 26.57) participated in the study. Women desired a slightly feminine voice, scoring 72.8 on the TWVQ, with mean pitch values of 165.2Hz on vowels and 144.5Hz in speech. Men desired a slightly masculine voice, scoring 68.4 on the TWVQ, with mean pitch values of 143.3Hz on vowels and 138.9Hz in speech. Of the seven evaluated measures, only the maximum pitch during number counting by women showed a moderate negative correlation with the TWVQ (p=0.043).

**Conclusion:**

Only maximum f_o_ during number counting by transgender women showed a negative correlation with the TWVQ score. Results suggest that although f_o_ may play a role in gender perception by voice, it is not the only determinant of vocal satisfaction in this population.

## INTRODUCTION

Human voice production is a complex phenomenon that involves physiological, psychological, and behavioral characteristics. Not only are these characteristics fundamental to communication, but they also play a crucial role in shaping an individual’s identity, conveying emotional, linguistic, and social nuances^(1,2)^.

Fundamental frequency, also known as oscillatory frequency^(3)^ (f_o_), defined as the number of vibratory cycles of the vocal folds in a time interval, is intrinsically linked to pitch, the auditory perception of frequencies that comprises bass and treble sounds^(4)^. In research contexts involving acoustic analysis of voice, f_o_ stands out as a predictor of specific characteristics related to speakers’ age and gender^(5)^. In Brazil, average f_o_ for cisgender adults ranges from 80Hz to 150Hz for men and 150Hz and 250Hz for women^(2)^, but the assumption that female voices are associated with high tones and male voices with low tones persists in the Brazilian culture^(6)^. Such an assumption bolsters a recurrent demand by transgender people in speech therapy clinics^(7,8)^, reflecting the social influence on the construction of gender norms and vocal expression. As a vehicle for gender expression, the voice is a complex element influenced by linguistic and communicative aspects, requiring a more comprehensive approach to understanding the transgender experience^(1)^.

In the context of gender diversity, f_o_ has been a prominent object of study and intervention in gender affirmation procedures. Particularly, trans individuals who identify with a gender other than the one assigned at birth^(9)^ often seek specialized support in the search for a voice aligned with their identity^(10)^. Hormone therapy, a common procedure in this context, triggers different effects in genders. Transgender women undergoing estrogen hormone therapy do not present evident voice transformations^(11)^, whereas transgender men experience f_o_ alterations due to muscle increase caused by testosterone, resulting in voice virilization^(12,13)^. But even with positive results from the testosterone effect, changes in pitch are not always enough for a voice to be considered as masculine by listeners^(14,15)^.

International studies highlight that despite changes in pitch and f_o_, the vocal satisfaction of transgender women may be more related to social acceptance of their voice than to f_o_ itself^(16,17)^. Voice satisfaction among trans men may be related to lower f_o_ values, although not all are satisfied with the voice changes promoted by hormone therapy alone^(18)^. This complex relation between f_o_ and vocal satisfaction, combined with the influence of cultural and linguistic factors, reinforces the need for investigations in populations such as the Brazilian one and the inclusion of transgender men in research on gender diversity.

Although voice studies constantly investigates f_o_ due to its robustness^(19)^, this research innovates by studying a set of f_o_ descriptors in a sample of Brazilian transgender men and women. It verified whether there are significant correlations between seven acoustic measures related to f_o_ position and variability and the vocal satisfaction of Brazilian transgender people.

## METHOD

A quantitative observational, cross-sectional study was submitted to and approved by the Research Ethics Committee, under opinion 4,730,175. Data collection was conducted from August 2021 to January 2022 at a school clinic in Campinas city, São Paulo, Brazil. All participants signed the informed consent form.

Participants were recruited via invitation on social networks, Facebook groups and e-mail for dissemination at LGBTQ+ reference centers, which configures a non-probabilistic sample by convenience.

Self-reporting transgender men and women aged from 18 to 49 years who were native Brazilian Portuguese speakers were invited to participate in the research. Age group delimitation was a methodological choice to avoid including people who had already gone through menopause in the sample, considering the possible resulting effects on the voice.

As exclusion criteria, the following was established: self-reported health problems that may affect voice quality on the day of data collection (e.g., flu, cold, gastroesophageal reflux or airway infections) and smoking. Additionally, participants should not self-report vocal health complaints on the day of data collection by answering the following question: “Do you have any voice health complaints?” Participants who reported dysphonia signs and symptoms such as hoarseness, vocal fatigue, pain and discomfort when speaking, among others, were excluded.

A total of 18 transgender people, 11 women and seven men, participated in the study. Participants were aged from 18 to 41 years, with a mean age of 26.91 for women and 26.57 for men. Fourteen participants were on hormone therapy, of which nine (81.81%) were trans women and five (71.43%) were trans men. None of the participants underwent gender affirmation vocal therapy prior to data collection. Table 1 presents the detailed characterization of the participants.

**Table 1:** Gender, age, profession, schooling, and information on surgical and hormonal procedures

**No.**	**Gender**	**Age**	**Laryngeal surgery**	**Hormone therapy**	**Age at hormone therapy initiation**	**Interruption?**
**1**	Trans man	21	No	Yes	18	Interrupted
**2**	Trans woman	30	No	No	–	–
**3**	Trans woman	40	No	Yes	38	No
**4**	Trans woman	22	No	Yes	19	No
**5**	Trans woman	41	No	Yes	37	15 days
**6**	Trans man	27	No	Yes	23	Interrupted
**7**	Trans woman	21	Chondroplasty	Yes	19	7 months
**8**	Trans woman	27	No	Yes	25	No
**9**	Trans woman	18	No	Yes	17	No
**10**	Trans man	23	No	No	–	–
**11**	Trans man	29	No	No	–	–
**12**	Trans woman	26	No	No	–	–
**13**	Trans man	18	No	Yes	17	1 month
**14**	Trans woman	18	No	Yes	19	2 months
**15**	Trans man	41	No	Yes	37	No
**16**	Trans woman	29	No	Yes	28	No
**17**	Trans man	27	No	Yes	26	No
**18**	Trans woman	24	No	Yes	24	No

### Procedures

Recording protocol consisted of uttering three sustained vowels [a], glissando using the vowel [a] and automatic speech (counting from one to ten). These tasks were selected to ensure production standardization among all study participants. Researchers demonstrated the tasks beforehand so the participants could practice and later the samples were recorded.

Production was recorded on a Dell Desktop computer using a unidirectional microphone Shure^®^ model SM58 coupled to a sound card Tascam^®^ model US100. Speakers were recorded directly by the Praat software (version 6.2.14)^(20)^ in mono channel, with a sampling rate of 44.1kHz and in .wav format. During the recording, participants were standing inside a soundproof booth with environmental noise below 50dB with the microphone positioned at a 90° angle ten centimeters from their mouths, as recommended by Dejonckere^(21)^.

The acoustic measurements f_o_ mean, f_o_ minimun (f_o_min) and f_o_ maximun (f_o_max) for the vowel samples; median (f_o_med), standard deviation (f_o_sd), f_o_ minimun (f_o_Min) and f_o_ maximum (f_o_max) for counting numbers were extracted and computed. These measurements were selected to understand the vocal range, predominant frequency and melodic variation of f_o_ in the voice and automatic speech samples.

Measurements of the sustained vowel [a] were extracted by the *get pich* function in Praat^(20)^, with windowing adjustment of the minimum and maximum parameters by gender (from 75 to 600 Hz^(22)^). Measurements related to number counting were extracted using the Prosody Descriptor script^(23)^. The script requires the audios to be tagged in Praat^(20)^, generating a TextGrid with the pauses and number of phonetic syllables identified in each speech excerpt. After its execution, the Prosody Descriptor script generated a text file report of all the extracted measurements which were then tabulated.

All participants answered the Transgender Woman Voice Questionnaire (TWVQ) originally proposed by Dacakis and Davies^(24)^, translated into and adapted for the Brazilian Portuguese by Santos and collaborators^(25)^. This 30-item instrument targets transgender women and addresses gender and voice satisfaction and its impact on daily life.

Each TWVQ question is scored on a four-point Likert scale according to the occurrence of the problem, where 1= never/rarely, 2= sometimes, 3= often, and 4= usually/always. Final score is calculated via simple summation, ranging from 30 to 120 points, and the higher the final score, the greater the impact of the voice on the respondent’s life. In addition to these 30 questions, the questionnaire has two final questions addressing individuals’ self-perception regarding gender expression in the voice: “currently my voice is” and “my ideal voice could sound,” which can be classified as ”very feminine,” “somewhat feminine,” “neutral,” “somewhat masculine,” and “very masculine.” We also adapted the questionnaire for application with trans men (Appendix A).

Descriptive and inferential analysis were performed using the Jamovi software, version 2.3.18. Shapiro-Wilk test showed that the samples are normal, thus comparison between groups was performed using Student’s t-test and the correlation of f_o_ measurements with the total TWVQ score was estimated using Pearson’s correlation. Significance level was set at 5%.

## RESULTS

Analysis of the means to the final TWVQ questions’ answers (Currently my voice is; My ideal voice should sound) showed that trans women consider their voice to be somewhat masculine (average score 4) and would like their voice to be more feminine (average score 2); whereas trans men consider their voice to be somewhat feminine (mean score 2) and would like their voice to be more masculine (average score 4). Table 2 summarizes the mean, minimum, maximum and standard deviation of the TWVQ scores and acoustic measurements extracted. Statistical analysis showed no differences between the groups.

**Table 2:** Descriptive and inferential analysis of quantitative variables by gender

	**Gender**	**Mean**	**Standard Deviation**	**Minimum**	**Maximum**	**Shapiro-Wilk**	**Student’s t**
						**p-value**	**p-value**
**TWVQ score**	M	68.4	21.16	34.00	100.0	0.899	0.665
	W	72.8	20.17	36.00	97.0	0.492	
**f_o_ mean (Hz)**	M	143.3	27.51	119.09	193.6	0.081	0.188
	W	165.2	35.78	110.60	208.4	0.091	
**f_o_ min - glissando (Hz)**	M	117.5	20.38	91.69	146.0	0.291	0.741
	W	121.6	27.35	85.42	170.7	0.199	
**f_o_ max - glissando (Hz)**	M	276.5	109.83	168.05	501.5	0.109	0.051
	W	373.4	84.99	200.32	475.2	0.215	
**f_o_ med numbers (Hz)**	M	138.9	23.04	106	180	0.758	0.671
	W	144.5	29.4	114	193	0.083	
**f_o_ sd numbers (Hz)**	M	17.0	8.04	8.51	30.4	0.21	0.709
	W	18.5	8.35	5.9	32.9	0.334	
**f_o_ min numbers (Hz)**	M	112.1	10.35	99	124	0.21	0.275
	W	124.1	26.46	92	173	0.119	
**f_o_ max numbers (Hz)**	M	203.7	55.5	137	286	0.547	0.639
	W	217.5	62.39	148	371	0.075	

**Caption:** f_o_ min = minimum value of f_o_; f_o_ max = maximum value of f_o_; f_o_ med = median of f_o_; f_o_ sd = standard deviation of f_o_; M = transgender man; W = Transgender woman

Table 3 shows the correlations between f_o_ measurements and the total TWVQ score, calculated using Pearson’s correlation coefficient. Importantly, interpreting these coefficients involves the parallelism of measurements on different scales. For the group of transgender women, we observed a moderate negative correlation between the maximum f_o_ number counting and the TWVQ (r = −0.584, p = 0.043).

**Table 3:** Correlation between each extracted acoustic measurement and the final TWVQ score for both genders

		**Men**	**Women**
**f_o_ mean**	R	0.167	−0.126
	p-value	0.72	0.712
**f_o_ min (glissando)**	R	0.118	−0.441
	p-value	0.8	0.175
**f_o_ max (glissando)**	R	0.085	−0.368
	p-value	0.856	0.266
**f_o_ med numbers**	R	0.318	−0.42
	p-value	0.487	0.199
**f_o_ sd numbers**	R	0.005	−0.33
	p-value	0.992	0.322
**f_o_ min numbers**	R	0.161	−0.081
	p-value	0.73	0.813
**f_o_ max numbers**	R	0.035	−0.617
	p-value	0.94	0.043*

Pearson’s Correlation Test

* p<0.05

**Caption:** f_o_ min = minimum value of f_o_; f_o_ max = maximum value of f_o_; f_o_ med = median of f_o_; f_o_ sd = standard deviation of f_o_; R = correlation coefficient

## DISCUSSION

Our study brings significant contributions to the field of gender and voice diversity research by exploring the correlation between f_o_ descriptors and vocal satisfaction among Brazilian transgender people. By considering a more comprehensive set of f_o_ descriptors, this study broadens the analysis to include transgender men, a population little explored in the scientific literature.

Results revealed that higher f_o_ values for trans women and lower f_o_ values for trans men had no direct relation with vocal satisfaction in the study sample, contrary to the common association between these values and gender expression in the voice^(26)^. Importantly, despite equal and unsatisfactory vocal perception, the mean f_o_ for the vowel [a] of trans men and women were within the expected ranges for the self-reported genders, but close to the limits of intersection between male and female voices^(2)^ known as the neutral range, between 145 and 175 Hz^(27)^. This definition is based on the results of studies derived from the project *Genderless voice* ^(27)^.

Although pitch is often associated with gender identification, gender and voice are heavily influenced by social factors^(5)^. A study conducted in Brazil observed that trans people, cisgender people, and speech therapists have different perceptions about gender expression in the voice^(28)^.

Regarding the self-perception of gender expression in the voice, both groups were dissatisfied as evidenced by the total TWVQ score (Table 2). Although the TWVQ protocol has yet to be validated in Brazil, the dissatisfaction found in our study is consistent with other recent studies conducted in the country^(29,30)^. Notably, the search for voices that are not extremely feminine nor masculine suggests a wider range of possibilities beyond the traditional gender dichotomy, indicating social influences that may contribute to trans women’s higher TWVQ scores.

One study^(29)^ found a positive relation between satisfaction with gender expression and Quality of Life in the voice of trans people and an average of 70.6 points in the total TWVQ score. Another study^(30)^ also noted a dissatisfaction of this population with their voice, showing mean TWVQ scores of 69.93 for men and 69.46 for women. Both studies assessed trans people’s self-perception without investigating the correlation with acoustics. Our mean score converged with the aforementioned findings. Additionally, none of the participants attended speech therapy, suggesting that they had not yet undergone specific interventions that influenced vocal satisfaction.

A study considering f_o_ analysis observed a negative correlation between the mean f_o_ of vowel [ɛ] and TWVQ scores in trans women^(31)^; however, this study disregarded other f_o_ descriptors and did not include trans men.

As for the extracted measurements, our analysis of the sustained vowel presented no sufficient information to correlate with vocal satisfaction (Table 3). However, maximum f_o_ during the automatic speech of trans women showed a moderate negative correlation with the TWVQ score, indicating that greater f_o_ variations during speech may contribute to greater voice satisfaction. Importantly, the difference between f_o_ values obtained by men and women during automatic speech was not statistically significant. This may be related to the difficulty that trans women have in maintaining the desired phonatory adjustments during speech when compared to vowel adjustments, for example^(32-34)^.

Our findings corroborate international studies which suggest that voice satisfaction in trans women is not strictly related to f_o,_ but rather to a combination of factors such as the other’s perception of their voice^(16,35)^. For the population studied, gender perception and vocal satisfaction may be influenced by Brazilian cultural and linguistic aspects.

Voice pitch and prosody are shaped by sociocultural practice and, thus, identifying characteristics that distinguish gender perception and theorizing from where gender differences in the voice come from must be closely linked to social factors^(5)^. But despite the particularities that the preferred voice assumes in different cultures, f_o_ alone, even with the analysis of its descriptors, cannot be associated with vocal satisfaction in transgender people and this should be considered in speech-language practice.

Transgender people’s satisfaction with their voice depends on the respective social acceptance^(5,16,17)^. Gender expression in the voice does not depend only on the speaker’s utterance, but also on the listener’s context^(28)^; thus, recent studies have discussed the need to consider a structure outside the binary in judging gender expression in the voice^(28,36,37)^. This study has some important limitations to be considered when interpreting the results. First, the absence of otorhinolaryngological evaluation required that all general health information be self-reported, but none of the participants had any complaints related to vocal health.

Second, the predominance of female participants may be related to the greater demand from this group for health services given the restricted results of hormone therapy^(11)^. Moreover, not all participants were undergoing hormone therapy, which may be related to the barriers to accessing health services such as discrimination and disrespect of social name^(38)^.

Finally, the use of automatic speech by number counting as an analysis sample may not represent the participants’ daily communication pattern but was an important methodological choice for standardizing data collection. New studies could benefit from spontaneous speech analysis, even with the standardization difficulties for all participants. We highlight the need for validating specific assessment and self-assessment instruments for this population, as well as developing a specific instrument for trans men. These limitations stress the need for caution when generalizing the present findings and highlight the importance of considering the participants’ social and individual context when analyzing their vocal characteristics.

Changes in the f_o_ alone may not guarantee full vocal satisfaction among transgender people. The need for training and improving individual communicative aspects combined with the promotion of visibility and social acceptance emerge as crucial aspects. Although speech therapy can offer benefits in the search for a more representative voice, the need for public policies and social measures aiming at promoting visibility and respect for the transgender population is evident, reflecting on the fullness of vocal satisfaction.

## CONCLUSION

Only maximum f_o_ during number counting by transgender women showed a moderate negative correlation with the TWVQ score. Research participants had mean f_o_ values within the expected ranges for the self-reported genders. TWVQ scores suggest dissatisfaction with the voice for both genders. Results demonstrate that although f_o_ may play a role in gender perception by voice, it was not determinant of vocal satisfaction in the studied population.

The complex relation between voice and gender identity transcends the voice’s physical characteristics and is strongly influenced by social, cultural, and individual factors. It is crucial to recognize that changes in fundamental frequency alone, even with hormone and surgical therapy, may not guarantee full vocal satisfaction for transgender people. Speech-language therapy follow-up is therefore crucial.

## Appendix A TWVQ adapted for transgender men

**Table:**

					**Rating Scale**					
					1 = never or rarely					
					2 = sometimes					
					3 = often					
					4 = usually or always					
Name: _____________________________________________________________________________						Date: ____________				
Based on your experience living as a man, please select the response that best applies to you.										
							**1**	**2**	**3**	**4**
1.	People have difficulty hearing me in a noisy room.						□	□	□	□
2.	I feel anxious when I know I have to use my voice						□	□	□	□
3.	My voice makes me feel less masculine than I would like.						□	□	□	□
4.	The pitch of my speaking voice is too high.						□	□	□	□
5.	The pitch of my voice is unreliable.						□	□	□	□
6.	My voice gets in the way of me living as a man.						□	□	□	□
7.	I avoid using the phone because of my voice.						□	□	□	□
8.	I’m tense when talking with others because of my voice.						□	□	□	□
9.	My voice gets croaky, hoarse or husky when I try to speak in a masculine voice.						□	□	□	□
10.	My voice makes it hard for me to be identified as a man.						□	□	□	□
11.	When I speak the pitch of my voice does not vary enough.						□	□	□	□
12.	I feel uncomfortable talking to friends, neighbours and relatives because of my voice.						□	□	□	□
13.	I avoid speaking in public because of my voice.						□	□	□	□
14.	My voice sounds artificial.						□	□	□	□
15.	I have to concentrate to make my voice sound the way I want it to sound.						□	□	□	□
16.	I feel frustrated with trying to change my voice.						□	□	□	□
17.	My voice difficulties restrict my social life.						□	□	□	□
18.	When I am not paying attention my pitch rises.						□	□	□	□
19.	When I laugh I sound like a woman.						□	□	□	□
20.	My voice doesn’t match my physical appearance.						□	□	□	□
21.	I use a great deal of effort to produce my voice.						□	□	□	□
22.	My voice gets tired quickly.						□	□	□	□
23.	My voice restricts the sort of work I do.						□	□	□	□
24.	I feel my voice does not reflect the ‘true me’.						□	□	□	□
25.	I am less outgoing because of my voice.						□	□	□	□
26.	I feel self-conscious about how strangers perceive my voice.						□	□	□	□
27.	My voice ‘gives out’ in the middle of speaking.						□	□	□	□
28.	It distresses me when I’m perceived as a woman because of my voice.						□	□	□	□
29.	The pitch range of my speaking voice is restricted.						□	□	□	□
30.	I feel discriminated against because of my voice.						□	□	□	□
										
*Please provide an overall rating of your voice:*										
*Currently, my voice is:*										
	□	□	□	□	□					
	Very feminine	Somewhat feminine	Gender neutral	Somewhat masculine	Very masculine					
*My ideal voice would sound:*										
	□	□	□	□	□					
	Very feminine	Somewhat feminine	Gender neutral	Somewhat masculine	Very masculine					

## References

[1] Santos LA, Antunes LB (2020). The social construction of the voice in gender performativity: a prosodic analysis in female transgender speech. Caletroscópio.

[2] Behlau M (2001). Voz: o livro do especialista..

[3] Švec JG, Schutte HK, Chen CJ, Titze IR (2023). Integrative insights into the myoelastic-aerodynamic theory and acoustics of phonation. scientific tribute to Donald G. Miller. J Voice.

[4] Sundberg J (2015). Ciência da voz: fatos sobre a voz na fala e no canto..

[5] Zimman L (2018). Transgender voices: insights on identity, embodiment, and the gender of the voice. Lang Linguist Compass.

[6] Braga A, Piovezani C (2021). Discursos sobre a fala feminina no Brasil contemporâneo. Rev ABRALIN..

[7] Sebastião TF, Constantini AC, Françozo MFC (2022). Transgender women: their narrative on health, voice, and dysphoria. Distúrb Comun.

[8] Constantini AC, Martinho DHC, Silva BAC, Lopes LW, Dornelas R, Ribeiro VV, Behlau M (2023). Identidade comunicativa: pessoas trans, travestis e não binárias..

[9] Jesus JG (2012). Orientações sobre identidade de gênero: conceitos e termos. Guia técnico sobre pessoas transexuais, travestis e demais transgêneros, para formadores de opinião.

[10] Granja MLS, Lucena JA, Silva MEF, Almeida TYN, Vasconcelos D, Araújo ANB, Pimentel BN (2021). Fundamentos científicos e prática clínica em fonoaudiologia..

[11] Schmidt JG, Goulart BNG, Dorfman MEKY, Kuhl G, Paniagua LM (2018). Voice challenge in transgender women: trans women self-perception of voice handicap as compared to gender perception of naïve listeners. Rev CEFAC.

[12] Hancock AB, Childs KD, Irwig MS (2017). Trans male voice in the first year of testosterone therapy: make no assumptions. J Speech Lang Hear Res.

[13] Deuster D, Matulat P, Knief A, Zitzmann M, Rosslau K, Szukaj M (2016). Voice deepening under testosterone treatment in female-to-male gender dysphoric individuals. Eur Arch Otorhinolaryngol.

[14] Thornton J (2008). Working with the transgender voice: the role of the speech and language therapist. Sexologies.

[15] Azul D (2015). Transmasculine people’s vocal situations: a critical review of gender-related discourses and empirical data. Int J Lang Commun Disord.

[16] McNeill EJM, Wilson JA, Clark S, Deakin J (2008). Perception of voice in the transgender client. J Voice.

[17] Hardy TLD, Rieger JM, Wells K, Boliek CA (2021). Associations between voice and gestural characteristics of transgender women and self-rated femininity, satisfaction, and quality of life. Am J Speech Lang Pathol.

[18] Nygren U, Nordenskjöld A, Arver S, Södersten M (2016). Effects on voice fundamental frequency and satisfaction with voice in trans men during testosterone treatment-a longitudinal study. J Voice.

[19] Behlau M, Almeida AA, Amorim G, Balata P, Bastos S, Cassol M (2022). Reducing the GAP between science and clinic: lessons from academia and professional practice - part A: perceptual-auditory judgment of vocal quality, acoustic vocal signal analysis and voice self-assessment. CoDAS.

[20] Boersma P, Weenink D (2022). Praat: doing phonetics by computer..

[21] Dejonckere PH, Bradley P, Clemente P, Cornut G, Crevier-Buchman L, Friedrich G (2001). A basic protocol for functional assessment of voice pathology, especially for investigating the efficacy of (phonosurgical) treatments and evaluating new assessment techniques: Guideline elaborated by the Committee on Phoniatrics of the European Laryngological Society (ELS). Eur Arch Otorhinolaryngol.

[22] Boersma P, Weenink D (2003). Sound: change gender.

[23] Barbosa PA (2016). “ProsodyDescriptorNew”..

[24] Dacakis G, Davies S, Oates JM, Douglas JM, Johnston JR (2013). Development and preliminary evaluation of the transsexual voice questionnaire for male-to-female transsexuals. J Voice.

[25] Santos HHANM, Aguiar AGO, Baeck HE, Van Borsel J (2015). Translation and preliminary evaluation of the brazilian portuguese version of the transgender voice questionnaire for male-to-female transsexuals. CoDAS.

[26] Cielo CA, Schwarz K, Gonçalves BFT, Lima JPM, Christmann MK (2021). Speech therapy for transgender women. Res Soc Dev.

[27] GenderLess Voice (2020). Meet Q: the first genderless voice.

[28] Martinho DHC, Constantini AC (2024). Auditory-perceptual assessment and acoustic analysis of gender expression in the voice. J Voice.

[29] Dornelas R, Guedes-Granzotti RB, Souza AS, Jesus AKB, Silva K (2020). Quality of life and voice: the vocal self-perception of transgender people. Audiol Commun Res.

[30] Santana EJ, Barbosa LJ, Irineu RA, Ribeiro VV (2022). Vocal self-perception of trans women and trans men. Res Soc Dev.

[31] Menezes DP, Lira ZS, Araújo ANB, Almeida AAF, Gomes AOC, Moraes BT (2024). Prosodic differences in the voices of transgender and cisgender women: self-perception of voice: an auditory and acoustic analysis. J Voice.

[32] Gelfer MP, van Dong BR (2013). A preliminary study on the use of vocal function exercises to improve voice in male-to-female transgender clients. J Voice.

[33] Quinn S, Swain N (2018). Efficacy of intensive voice feminisation therapy in a transgender young offender. J Commun Disord.

[34] Gelfer MP, Tice RM (2013). Perceptual and acoustic outcomes of voice therapy for male-to-female transgender individuals immediately after therapy and 15 months later. J Voice.

[35] Hancock AB, Krissinger J, Owen K (2011). Voice perceptions and quality of life of transgender people. J Voice.

[36] Merritt B, Bent T, Kilgore R, Eads C (2024). Auditory free classification of gender diverse speakers. J Acoust Soc Am.

[37] Hardy TLD, Boliek CA, Wells K, Dearden C, Zalmanowitz C, Rieger JM (2016). Pretreatment acoustic predictors of gender, femininity, and naturalness ratings in individuals with male-to-female gender identity. Am J Speech Lang Pathol.

[38] Rocon PC, Sodré F, Rodrigues A, Barros MEB, Wandekoken KD (2019). Challenges faced by transgender people in accessing the transexualizer process of the Brazilian National Health System. Interface Commun Heal Educ..

