# Supplementary material for: Different measures of fundamental frequency and vocal satisfaction among transgender men and women
Source: Codas. 2025 Jan 27;37(1):e20240087. doi: 10.1590/2317-1782/e20240087en (PMC11781363; doi:10.1590/2317-1782/e20240087en)
Supplement: Translation

# Translation: Diferentes medidas de frequência fundamental e satisfação vocal de homens e mulheres transgênero

**DOI:** 10.1590/2317-1782/e20240087pt

**Authors:** Diego Henrique da Cruz Martinho, Eric Rodrigues Dias, Ana Carolina Constantini

**Affiliations:** 1 Departamento de Saúde Interdisciplinaridade e Reabilitação, Faculdade de Ciências Médicas, Universidade Estadual de Campinas – UNICAMP - Campinas (SP), Brasil.; 2 Programas de Residência Multiprofissional, Hospital das Clínicas, Faculdade de Medicina, Universidade de São Paulo – USP - São Paulo (SP), Brasil.

**Keywords:** Voz, Treinamento da Voz, Acústica da Fala, Identidade de Gênero, Comunicação

## Abstract

**Objetivo:**

Verificar se existe relação entre medidas acústicas relacionadas f_o_ e a satisfação vocal de pessoas transgênero brasileiras.

**Método:**

Estudo quantitativo observacional e transversal, com aplicação do Trans Woman Voice Questionnaire (TWVQ), gravação das vozes (vogal sustentada e fala automática) e extração de sete medidas acústicas relacionadas à posição e variabilidade da f_o_ de pessoas transgênero. Participantes divididos em dois grupos de acordo com o gênero. Realizada análise descritiva e inferencial, a comparação entre os grupos foi realizada pelo Teste T de Student e a correlação das medidas da f_o_ com o protocolo TWVQ foi testada por meio da Correlação de Pearson (p<0,05).

**Resultados:**

Participaram 11 mulheres (média de idade= 26,91) e sete homens transgênero (média de idade = 26,57). As mulheres desejavam uma voz um pouco feminina, pontuando 72,8 no TWVQ, com valores médios de f_o_ de 165,2Hz nas vogais e 144,5Hz na fala. Os homens desejavam uma voz um pouco masculina, pontuando 68,4 no TWVQ, com valores médios de f_o_ de 143,3Hz nas vogais e 138,9Hz na fala. Dentre as sete medidas avaliadas, apenas f_o_ máxima durante a contagem de números nas mulheres apresentou correlação negativa moderada com o TWVQ (p=0,043).

**Conclusão:**

Apenas a f_o_ máxima durante a contagem de números das mulheres transgênero demonstrou uma correlação negativa com o escore do TWVQ. Os resultados obtidos sugerem que, embora a f_o_ possa desempenhar um papel na percepção do gênero na voz, ela não é o único determinante da satisfação vocal nesta população.

## INTRODUÇÃO

A produção da voz humana é um fenômeno complexo que envolve características fisiológicas, psicológicas e comportamentais. Essas características não apenas são fundamentais para a comunicação, como também desempenham um papel crucial na formação da identidade do indivíduo, transmitindo nuances emocionais, linguísticas e sociais^(1,2)^.

A frequência fundamental, também conhecida como frequência oscilatória^(3)^ (f_o_), é definida como a quantidade de ciclos vibratórios das pregas vocais em um intervalo de tempo, e está intrinsecamente ligada ao *pitch*, que é a percepção auditiva de frequências que compreende sons graves e agudos^(4)^. Em contextos de pesquisa que envolvem a análise acústica da voz, a medida da f_o_ frequentemente se destaca como um preditor de características específicas relacionadas à idade e gênero dos falantes^(5)^. No Brasil a média de f_o_ para adultos cisgêneros varia entre 80Hz a 150Hz para os homens e 150Hz e 250Hz para mulheres^(2)^.Contudo, persiste na cultura brasileira a concepção de que vozes femininas são associadas a tons agudos e as masculinas a tons graves^(6)^. Esta percepção alimenta uma demanda recorrente de pessoas transgênero nas clínicas fonoaudiológicas^(7,8)^, refletindo a influência social na construção de padrões de gênero e na expressão vocal. A voz, como veículo de expressão do gênero, é um elemento complexo influenciado por aspectos linguísticos e comunicativos, exigindo uma abordagem mais abrangente na compreensão da transgeneridade^(1)^.

No âmbito da diversidade de gênero, a f_o_ tem sido um objeto de estudo e de intervenção proeminente nos procedimentos de afirmação de gênero. Em particular, pessoas transgênero (ou trans), que se identificam com um gênero diferente do atribuído ao nascimento^(9)^, procuram frequentemente o suporte especializado na busca por uma voz alinhada à sua identidade^(10)^. A terapia hormonal, procedimento comum nesse contexto, desencadeia diferentes efeitos entre os gêneros. Mulheres transgênero, submetidas à terapia hormonal com estrogênio, não apresentam transformações evidentes na voz^(11)^, enquanto que em homens transgênero ocorrem alterações na f_o_ devido ao aumento muscular provocado pela testosterona, resultando na virilização da voz^(12,13)^. Entretanto, mesmo com resultados positivos ao efeito da testosterona, nem sempre a mudança do *pitch* é suficiente para que uma voz seja considerada pelos ouvintes como masculina^(14,15)^.

Estudos internacionais destacam que mesmo que haja mudanças na f_o_ e no *pitch*, a satisfação vocal de mulheres transgênero pode estar mais relacionada à aceitação social da voz do que à própria frequência fundamental^(16,17)^. Para os homens trans, a satisfação com a voz pode ter relação com valores mais baixos de f_o_, apesar de nem todos os homens trans se sentirem satisfeitos com as mudanças na voz promovidas apenas pela terapia hormonal^(18)^. Essa complexidade na relação entre f_o_ e satisfação vocal, aliada à influência de fatores culturais e linguísticos, reforça a necessidade de investigações em outras populações como a brasileira e a inclusão dos homens transgênero nas pesquisas sobre a diversidade de gênero.

Apesar da f_o_ ser constantemente investigada nos estudos da área da voz devido a sua robustez^(19)^, esta pesquisa inova ao estudar um conjunto de descritores dessa medida em uma amostra de pessoas brasileiras, além de considerar a análise de homens transgênero, que são menos explorados nesse contexto. Deste modo, o objetivo deste estudo é verificar se existem relações significativas entre sete medidas acústicas relacionadas à posição e variabilidade da f_o_ e a satisfação vocal de pessoas transgênero brasileiras.

## MÉTODO

Trata-se de estudo quantitativo observacional, transversal submetido e aprovado pelo Comitê de Ética em Pesquisa, sob parecer 4.730.175. A coleta de dados foi realizada no período de agosto de 2021 a janeiro de 2022 em uma clínica escola da cidade de Campinas, São Paulo, Brasil. Todos os indivíduos envolvidos assinaram o Termo de Consentimento Livre e Esclarecido.

Os participantes foram recrutados por meio de divulgação em redes sociais, grupos de Facebook e e-mail, com convite para divulgação em centros de referência LGBTQ+, o que configura uma composição de amostra não-probabilística, por conveniência.

Foram convidados a participar da pesquisa pessoas que se autorrelataram homens e mulheres transgênero que tivessem entre 18 anos e 49 anos e fossem falantes nativos de português brasileiro. A faixa etária foi uma escolha metodológica para que a amostra não incluísse pessoas que já tivessem passado pela menopausa, considerando os possíveis efeitos na voz decorrentes desse período.

Os critérios de exclusão foram: autorrelatar problemas de saúde que possam afetar a qualidade vocal no dia da coleta como gripe, resfriado, refluxo gastroesofágico ou infecções de vias aéreas; e tabagismo. Além disso, o participante não devia autorrelatar queixas de saúde vocal no dia da coleta, para essa informação o participante respondia a seguinte questão: “Você tem alguma queixa sobre a saúde da sua voz?”, os participantes que relatassem sinais e sintomas de disfonia como rouquidão, fadiga vocal, dor e desconforto ao falar, entre outros, não foram incluídos na pesquisa.

Participaram do estudo 18 pessoas transgênero, sendo 11 mulheres e sete homens. Os participantes tinham entre 18 e 41 anos sendo que a média da idade foi de 26,91 para as mulheres e 26,57 para os homens. Catorze participantes estavam em terapia hormonal, sendo nove (81,81%) das mulheres transgênero e cinco (71,43%) dos homens transgênero. Nenhum dos participantes realizou terapia vocal para afirmação de gênero prévia à coleta de dados. A Tabela 1 mostra a caracterização detalhada dos participantes.

**Tabela 1:** Gênero, idade, profissão, escolaridade e informações sobre procedimentos cirúrgicos e hormonais

**Nº**	**Gênero**	**Idade**	**Cirurgia laríngea**	**Hormonoterapia**	**Idade de início da hormonioterapia**	**Interrupção?**
**1**	Homem trans	21	Não	Sim	18	Interrompeu
**2**	Mulher trans	30	Não	Não	-	-
**3**	Mulher trans	40	Não	Sim	38	Não
**4**	Mulher trans	22	Não	Sim	19	Não
**5**	Mulher trans	41	Não	Sim	37	15 dias
**6**	Homem trans	27	Não	Sim	23	Interrompeu
**7**	Mulher trans	21	Condroplastia	Sim	19	7 meses
**8**	Mulher trans	27	Não	Sim	25	Não
**9**	Mulher trans	18	Não	Sim	17	Não
**10**	Homem trans	23	Não	Não	-	-
**11**	Homem trans	29	Não	Não	-	-
**12**	Mulher trans	26	Não	Não	-	-
**13**	Homem trans	18	Não	Sim	17	1 mês
**14**	Mulher trans	18	Não	Sim	19	2 meses
**15**	Homem trans	41	Não	Sim	37	Não
**16**	Mulher trans	29	Não	Sim	28	Não
**17**	Homem trans	27	Não	Sim	26	Não
**18**	Mulher trans	24	Não	Sim	24	Não

### Procedimentos

O protocolo de gravação consistia na emissão de três vogais [a] sustentadas, emissão em glissando utilizando a vogal [a] e fala automática (contagem de números de um a dez). Estas tarefas foram selecionadas como escolha metodológica para garantir a padronização de produção entre todos os participantes do estudo. As tarefas foram demonstradas pelos pesquisadores previamente antes da gravação, os participantes puderam praticar a execução e posteriormente as amostras foram gravadas.

A gravação foi realizada com o auxílio de um computador Desktop Dell, um microfone unidirecional da marca Shure® modelo SM58, acoplado a uma placa de som da marca Tascam® modelo US100. Os falantes foram gravados diretamente pelo software Praat (versão 6.2.14)^(20)^ em mono canal, com taxa de amostragem de 44,1kHz e em formato .wav. Durante a gravação o participante encontrava-se em pé, dentro de uma cabina acústica com ruído ambiental abaixo de 50dB, o microfone foi posicionado em um ângulo de 90º a 10 centímetros da boca do participante, conforme preconizado por Dejonckere^(21)^.

Foi realizada a extração e análise automatizada das seguintes medidas acústicas da frequência fundamental: f_o_ média, f_o_ mínima (f_o_min) e f_o_ máxima (f_o_máx) para as amostras de vogais; mediana de f_o_ (f_o_med), desvio-padrão de f_o_ (f_o_sd), f_o_ mínima (f_o_min) e f_o_ máxima (f_o_máx) para a contagem de números. A escolha de tais medidas se deu com o objetivo de compreender a extensão vocal, frequência predominante e a variação melódica de f_o_ nas amostras de voz e fala automática da amostra estudada.

A extração das medidas da vogal [a] sustentada foi realizada por meio da função *get pitch* no Praat^(20)^ com ajuste de janelamento dos parâmetros de mínimo e máximo conforme o gênero (entre 75 e 600Hz^(22)^). A extração das medidas referentes à contagem de números foi realizada por meio do script Prosody Descriptor^(23)^. O script demanda que os áudios sejam etiquetados no Praat^(20)^, gerando um TextGrid com a identificação das pausas e do número de sílabas fonéticas em cada trecho de fala. Após sua execução, o script Prosody Descriptor gerou um relatório em arquivo de texto de todas as medidas extraídas e estes dados foram tabulados.

Os participantes responderam ao Transgender Woman Voice Questionnaire (TWVQ) proposto originalmente por Dacakis e Davies^(24)^, traduzido e adaptado para o português brasileiro por Santos e colaboradores^(25)^, este questionário é direcionado a mulheres transgênero e possui 30 questões que abordam a satisfação com o gênero e a voz e seu impacto no cotidiano.

Cada questão do TWVQ pode ser pontuada em uma escala likert de quatro pontos de acordo com a ocorrência do problema, em que 1= nunca/raramente, 2= algumas vezes, 3= frequentemente, e 4= usualmente/sempre. O escore final do protocolo é calculado via somatória simples, variando entre 30 a 120 pontos, sendo que quanto maior o escore final, maior o impacto da voz na vida do respondente. Além destas 30 questões, o questionário possui duas questões finais abordando a autopercepção do sujeito quanto à expressão do gênero na voz: “atualmente minha voz é” e “minha voz ideal poderia soar”, podendo ser classificada em “muito feminina”, “um pouco feminina”, “neutra”, “um pouco masculina” e “muito masculina”. O questionário foi adaptado pelos pesquisadores para ser aplicado também em pessoas que performam papeis do gênero masculino (Apêndice A).

Os resultados passaram por análise estatística descritiva e inferencial por meio do *software* Jamovi versão 2.3.18. O teste de Shapiro-Wilk evidenciou que as amostras são normais e, deste modo, a comparação entre os grupos foi realizada por meio do Teste T de Student e a correlação das medidas da f_o_ com o escore total do protocolo TWVQ foi testada por meio da Correlação de Pearson. Foi adotado um nível de significância de 5%.

## RESULTADOS

A análise das médias das respostas das perguntas finais do TWVQ (Atualmente minha voz é; Minha voz ideal deveria soar) mostrou que as mulheres transgênero consideram a voz um pouco masculina (escore médio 4) e gostariam que a voz fosse um pouco feminina (escore médio 2); e os homens transgênero consideram a voz um pouco feminina (escore médio 2) e gostariam que a voz fosse um pouco masculina (escore médio 4). A Tabela 2 evidencia os resultados da pontuação média, mínimo, máximo e desvio padrão dos escores do TWVQ e das medidas acústicas extraídas. A análise estatística não evidenciou diferenças entre os grupos.

**Tabela 2:** Análise descritiva e inferencial das variáveis quantitativas por gênero

	**Gênero**	**Média**	**Desvio-padrão**	**Mínimo**	**Máximo**	**Shapiro-Wilk**	**T de Student**
						**p-valor**	**p-valor**
**TWVQ escore**	H	68,4	21,16	34	100	0,899	0,665
	M	72,8	20,17	36	97	0,492	
**f_o_ média (Hz)**	H	143,3	27,51	119,09	193,6	0,081	0,188
	M	165,2	35,78	110,6	208,4	0,091	
**f_o_ min - glissando (Hz)**	H	117,5	20,38	91,69	146	0,291	0,741
	M	121,6	27,35	85,42	170,7	0,199	
**f_o_máx – glissando (Hz)**	H	276,5	109,83	168,05	501,5	0,109	0,051
	M	373,4	84,99	200,32	475,2	0,215	
**Números f_o_med (Hz)**	H	138,9	23,04	106	180	0,758	0,671
	M	144,5	29,4	114	193	0,083	
**Números f_o_sd (Hz)**	H	17	8,04	8,51	30,4	0,21	0,709
	M	18,5	8,35	5,9	32,9	0,334	
**Números f_o_min (Hz)**	H	112,1	10,35	99	124	0,21	0,275
	M	124,1	26,46	92	173	0,119	
**Números f_o_max (Hz)**	H	203,7	55,5	137	286	0,547	0,639
	M	217,5	62,39	148	371	0,075	

**Legenda:** f_o_min = valor mínimo de f_o_; f_o_máx = valor máximo de f_o_; f_o_med = mediana de f_o_; f_o_sd = desvio padrão de f_o_; H = homem transgênero; M = Mulher transgênero

A Tabela 3 mostra as correlações entre as medidas de f_o_ e o escore total do TWVQ, calculadas usando o coeficiente de correlação de Pearson. É importante destacar que a interpretação desses coeficientes envolve o paralelismo de medidas em escalas diferentes. Para o grupo de mulheres transgênero, observou-se uma correlação negativa moderada entre a f_o_ máxima na contagem de números e o TWVQ (r = -0,584, p = 0,043).

**Tabela 3:** Correlação entre cada medida acústica extraída e o escore final do protocolo TWVQ para ambos os gêneros

		**Homens**	**Mulheres**
**f_o_média**	R	0,167	-0,126
	p-valor	0,72	0,712
**f_o_min (glissando)**	R	0,118	-0,441
	p-valor	0,8	0,175
**f_o_máx (glissando)**	R	0,085	-0,368
	p-valor	0,856	0,266
**Números f_o_med**	R	0,318	-0,42
	p-valor	0,487	0,199
**Números f_o_sd**	R	0,005	-0,33
	p-valor	0,992	0,322
**Números f_o_min**	R	0,161	-0,081
	p-valor	0,73	0,813
**Números f_o_max**	R	0,035	-0,617
	p-valor	0,94	0,043*

Teste de Correlação de Pearson

* p<0.05

**Legenda:** f_o_min = valor mínimo de f_o_; f_o_máx = valor máximo de f_o_; f_o_med = mediana de f_o_; f_o_sd = desvio padrão de f_o_; R = Coeficiente de Correlação

## DISCUSSÃO

Este estudo traz contribuições significativas para o campo da pesquisa em diversidade de gênero e voz ao explorar a relação entre descritores de f_o_ e a satisfação vocal em pessoas transgênero brasileiras. Ao considerar um conjunto mais abrangente de descritores da f_o_, este estudo amplia a análise para incluir homens transgênero, uma população menos explorada na literatura científica.

A análise dos resultados revelou que valores mais altos da frequência fundamental (f_o_) para mulheres e mais baixos para homens transgêneros não demonstraram uma relação direta com a satisfação vocal nas pessoas estudadas, contrariando a associação comum entre esses valores e a percepção de gênero na voz^(26)^. Uma observação importante é que, apesar da percepção vocal igual e insatisfatória, as médias de f_o_ da vogal [a] de homens e mulheres trans estavam dentro das faixas esperadas para os gêneros autorreferidos^(2)^, porém, próximas aos limites de interseção entre vozes masculinas e femininas, conhecida como faixa neutra, entre 145 e 175Hz^(27)^. Esta definição tem como base os resultados de estudos derivados de um projeto chamado *Genderless voice* ^(27)^.

Embora o *pitch* vocal seja frequentemente associado à identificação do gênero, é notável que gênero e voz são fortemente influenciados por fatores sociais^(5)^. Um estudo realizado no Brasil evidenciou que pessoas trans, cisgênero e fonoaudiólogos têm percepções diferentes sobre a expressão do gênero pela voz^(28)^.

Quanto à autopercepção da expressão do gênero na voz no presente estudo, ambos os grupos de participantes apresentaram insatisfação, conforme evidenciado pelos escores totais do protocolo TWVQ evidenciados na Tabela 2. Embora o protocolo ainda não esteja validado no Brasil (apesar de ser amplamente utilizado), a insatisfação encontrada neste estudo é consistente com outros estudos recentes realizados no país^(29,30)^. Notavelmente, a busca por vozes que não sejam extremamente femininas ou masculinas sugere uma gama mais ampla de possibilidades, além da tradicional dicotomia de gênero, indicando influências sociais que podem contribuir para as pontuações mais altas das mulheres trans no TWVQ.

Um estudo^(29)^ evidenciou relação positiva entre a satisfação com o gênero na voz e a Qualidade de Vida em Voz de pessoas transgênero e encontrou média 70,6 pontos no escore total TWVQ. Outro estudo^(30)^ também mostrou a insatisfação dessa população com a voz evidenciando médias no TWVQ de 69,93 para os homens e, 69,46 para as mulheres. Ambos os estudos avaliaram a autopercepção de pessoas trans e não tinham como foco a correlação com a acústica. O escore médio obtido em nossa pesquisa convergiu com os achados dos estudos supracitados. Além disso, é relevante observar que nenhum dos participantes estava em terapia fonoaudiológica, sugerindo que ainda não haviam realizado intervenções específicas que influenciassem na satisfação vocal.

Um estudo que considerou a análise da f_o_ observou correlação negativa entre a f_o_ média da vogal [ɛ] e escores do TWVQ em mulheres trans^(31)^, entretanto este estudo não considera outros descritores de f_o_ e não incluiu homens transgênero.

No contexto das medidas extraídas, a análise da vogal sustentada não apresentou informações suficientes para correlação com a satisfação vocal, conforme demonstrado na Tabela 3. Contudo, a f_o_ máxima durante a fala automática das mulheres transgênero evidenciou uma correlação negativa moderada com o questionário TWVQ, indicando que maiores variações de f_o_ durante a fala dessas mulheres podem contribuir para uma maior satisfação. Destaca-se que, a diferença da f_o_ entre os valores obtidos por homens e mulheres na fala automática não foi significativo. Esse dado pode ter relação com a dificuldade que mulheres trans têm em manter os ajustes fonatórios desejados durante a fala quando comparados aos ajustes da vogal, por exemplo^(32-34)^.

Nossos achados corroboram estudos internacionais que sugerem que a satisfação com a voz em mulheres trans não está estritamente relacionada à f_o_, mas, sim, a uma combinação de fatores, como a percepção do outro sobre a sua voz^(16,35)^. Ressalta-se que para a população estudada, a percepção de gênero e a satisfação vocal podem ser influenciadas por aspectos culturais e linguísticos no Brasil.

O *pitch* da voz e a forma de falar são moldados pela prática sociocultural e, deste modo, a identificação de características que distinguem a percepção do gênero e a teorização de onde vêm as diferenças de gênero na voz devem estar intimamente ligados à fatores sociais^(5)^, entretanto, mesmo com as particularidades que a voz preferida assume em diferentes culturas, somente a f_o_, mesmo com a análise também de seus descritores, não pode ser associada com satisfação vocal em pessoas transgênero e isso deve ser considerado na prática clínica fonoaudiológica.

A satisfação de pessoas transgênero com a voz tem relação com a aceitação social de suas vozes^(5,16,17)^. É evidente que a percepção do gênero na voz não depende somente da emissão do falante, mas também do contexto do ouvinte^(28)^, deste modo, estudos recentes têm discutido a necessidade de se considerar uma estrutura diferente da binária no julgamento do gênero na voz^(28,36,37)^.Este estudo apresenta algumas limitações importantes que devem ser consideradas ao interpretar os resultados. Primeiramente, a ausência de avaliação otorrinolaringológica fez necessária que todas as informações de saúde geral fossem autorreferidas, entretanto, nenhum dos participantes tinha qualquer queixa relativa à saúde vocal.

A predominância de participantes do gênero feminino na pesquisa pode ter relação com a maior procura destas pessoas por serviços de saúde, visto os resultados limitados da terapia homonal^(11)^. Além disso, é crucial destacar que nem todos os participantes estavam realizando terapia hormonal, fato que pode estar relacionado às barreiras que essa população encontra ao acessar os serviços de saúde como discriminação e desrespeito ao nome social^(38)^.

Outra limitação é o uso da fala automática por meio da contagem de números como amostra de análise, o que pode não representar o padrão de comunicação cotidiano dos participantes, mas foi importante como escolha metodológica de padronização da coleta de dados. Novos estudos podem se beneficiar de uma análise de fala espontânea, mesmo com as dificuldades de padronização para todos os participantes. Destaca-se a necessidade de validação de instrumentos de avaliação e autoavaliação específicos para essa população, bem como o desenvolvimento de um instrumento específico para homens trans. Essas limitações ressaltam a necessidade de cautela ao generalizar os achados deste estudo e destacam a importância de considerar o contexto social e individual dos participantes ao analisar suas características vocais.

O estudo evidencia que mudanças apenas na f_o_, podem não garantir a plena satisfação vocal de pessoas transgênero. A necessidade de treinamento e aperfeiçoamento de aspectos comunicativos individuais, aliada à promoção da visibilidade e aceitação social, emergem como aspectos cruciais. Assim, embora a terapia fonoaudiológica possa oferecer benefícios na busca por uma voz mais representativa, é evidente a necessidade de políticas públicas e medidas sociais que visem à visibilidade e respeito da população transgênero, refletindo na plenitude da satisfação vocal.

## CONCLUSÃO

Apenas a f_o_ máxima durante a contagem de números das mulheres transgênero demonstrou correlação negativa moderada com o escore do TWVQ. Os participantes do estudo apresentaram valores médios de f_o_ dentro das faixas esperadas para os gêneros autorreferidos e os escores do TWVQ sugerem insatisfação com a voz para ambos os gêneros. Os resultados obtidos evidenciam que, embora a frequência fundamental possa desempenhar um papel na percepção do gênero na voz, ela não foi determinante da satisfação vocal na amostra estudada.

A complexidade da relação entre a voz e a identidade de gênero transcende as características físicas da voz, sendo fortemente influenciada por fatores sociais, culturais e individuais. É crucial reconhecer que mudanças apenas na frequência fundamental, mesmo com terapia hormonal e cirúrgica, podem não garantir a plena satisfação vocal das pessoas transgênero. Portanto, o acompanhamento fonoaudiológico é crucial.

## Apêndice A TWVQ adaptado para homens transgênero

**Table:**

					**Escala de classificação**					
					1 = nunca ou raramente					
					2 = algumas vezes					
					3 = frequentemente					
					4 = usualmente ou sempre					
Nome: _____________________________________________________________________________						Data: ____________				
Com base na sua experiência em viver como homem, assinale, por favor, a resposta que mais se adeque a você.										
							**1**	**2**	**3**	**4**
1.	As pessoas têm dificuldade em me ouvir em uma sala barulhenta.						□	□	□	□
2.	Eu me sinto ansioso quando sei que tenho que usar minha voz.						□	□	□	□
3.	Minha voz faz com que eu me sinta menos masculino do que eu gostaria.						□	□	□	□
4.	O tom da minha voz falada é muito agudo.						□	□	□	□
5.	É difícil saber como sairá o tom da minha voz.						□	□	□	□
6.	Minha voz atrapalha a minha vida como homem.						□	□	□	□
7.	Eu evito usar o telefone por causa da minha voz.						□	□	□	□
8.	Eu fico tenso quando falo com os outros por causa da minha voz.						□	□	□	□
9.	Fico rouco quando tento falar com minha voz masculina.						□	□	□	□
10.	Minha voz dificulta que eu seja reconhecido como homem.						□	□	□	□
11.	A variação de tons da minha voz masculina é pequena.						□	□	□	□
12.	Eu me sinto desconfortável ao falar com amigos, vizinhos ou parentes por causa da minha voz.						□	□	□	□
13.	Eu evito falar em público por causa da minha voz.						□	□	□	□
14.	Minha voz parece artificial.						□	□	□	□
15.	Tenho que me concentrar para fazer minha voz soar como quero que soe.						□	□	□	□
16.	Eu me sinto frustrado quando tento mudar a minha voz.						□	□	□	□
17.	As dificuldades com a minha voz restringem a minha vida social.						□	□	□	□
18.	Quando eu não estou prestando atenção meu tom de voz fica agudo.						□	□	□	□
19.	Quando eu rio pareço uma mulher.						□	□	□	□
20.	Minha voz não combina com minha aparência física.						□	□	□	□
21.	Eu faço muito esforço para produzir minha voz.						□	□	□	□
22.	Minha voz fica cansada rapidamente.						□	□	□	□
23.	Minha voz restringe o tipo de trabalho que faço.						□	□	□	□
24.	Eu sinto que a minha voz não reflete o meu ‘verdadeiro eu’.						□	□	□	□
25.	Eu sou menos extrovertido por causa da minha voz.						□	□	□	□
26.	Eu tenho consciência sobre como os estranhos percebem minha voz.						□	□	□	□
27.	Minha voz ‘falha’ no meio da fala.						□	□	□	□
28.	Fico aborrecido quando sou percebido como uma mulher por causa da minha voz.						□	□	□	□
29.	Minha voz falada possui uma variação de tons pequena.						□	□	□	□
30.	Eu me sinto discriminado por causa da minha voz.						□	□	□	□
										
*Por favor, forneça uma avaliação global da sua voz:*										
*Atualmente minha voz é:*										
	□	□	□	□	□					
	Muito feminina	Um pouco feminina	Neutra	Um pouco masculina	Muito masculina					
*Minha voz ideal poderia soar:*										
	□	□	□	□	□					
	Muito feminina	Um pouco feminina	Neutra	Um pouco masculina	Muito masculina
